# Temporal and spatial variability of dynamic microstate brain network in early Parkinson’s disease

**DOI:** 10.1038/s41531-023-00498-w

**Published:** 2023-04-10

**Authors:** Chunguang Chu, Zhen Zhang, Jiang Wang, Zhen Li, Xiao Shen, Xiaoxuan Han, Lipeng Bai, Chen Liu, Xiaodong Zhu

**Affiliations:** 1 School of Electrical and Information Engineering, Tianjin University, 300072 Tianjin, China; 2 Department of Neurology, Tianjin Neurological Institute, Tianjin Medical University General Hospital, 300052 Tianjin, China

**Keywords:** Parkinson's disease, Diagnostic markers

## Abstract

Changes of brain network dynamics reveal variations in macroscopic neural activity patterns in behavioral and cognitive aspects. Quantification and application of changed dynamics in brain functional connectivity networks may contribute to a better understanding of brain diseases, and ultimately provide better prognostic indicators or auxiliary diagnostic tools. At present, most studies are focused on the properties of brain functional connectivity network constructed by sliding window method. However, few studies have explored evidence-based brain network construction algorithms that reflect disease specificity. In this work, we first proposed a novel approach to characterize the spatiotemporal variability of dynamic functional connectivity networks based on electroencephalography (EEG) microstate, and then developed a classification framework for integrating spatiotemporal variability of brain networks to improve early Parkinson’s disease (PD) diagnostic performance. The experimental results indicated that compared with the brain network construction method based on conventional sliding window, the proposed method significantly improved the performance of early PD recognition, demonstrating that the dynamic spatiotemporal variability of microstate-based brain networks can reflect the pathological changes in the early PD brain. Furthermore, we observed that the spatiotemporal variability of early PD brain network has a specific distribution pattern in brain regions, which can be quantified as the degree of motor and cognitive impairment, respectively. Our work offers innovative methodological support for future research on brain network, and provides deeper insights into the spatiotemporal interaction patterns of brain activity and their variabilities in early PD.

## Introduction

Parkinson’s disease (PD) is a common neurodegenerative disorder, and the main clinical manifestations are motor symptoms (e.g., quiescent tremor, bradykinesia, or myotonia) and non-motor symptoms (e.g., cognitive impairment)^1^. The most important pathological change of PD is the degeneration and death of dopaminergic neurons in the substantia nigra, which leads to a significant decrease of dopamine content in the striatum^2^. Even in the early stage of PD, the death of dopaminergic neurons in the substantia nigra is at least 50% and the dopamine content in the striatum is 80% or more reduced^2^. However, the clinical symptoms of early PD are not typical and reliable clinical indicators are lacking, which seriously affects the timely diagnosis and treatment of PD. In recent years, numerous studies have tried to find early biomarkers to evaluate PD risk in a rapid and rigorous way. Moreover, the early treatment of PD may delay the disease progression and prevent some non-motor function impairment of brain. Therefore, it is of great significance to reveal the abnormal brain activity manifestations of early PD and further provide an accurate diagnosis tool for it.

The human brain can be regarded as a network composed of a large number of neurons through mutual synaptic connections. Dopamine deficiency is associated with pathological and compensatory changes in the organization and connectivity of brain networks^3–5^, affecting several broadly distributed neural circuits^6^. Resting state electroencephalography (EEG) is the output of neural electrical activity caused by brain physiological function^7^ and has high temporal resolution and standard spatial distribution. Therefore, resting state EEG has been widely used to describe the spatiotemporal electrical activities of the whole brain and reveal the functional connectivity networks of scalp. Many studies have applied functional connectivity networks to the analysis of dyskinesia and cognitive impairment in PD via group analysis, and reported a series of abnormal network properties. For example, Hassan et al.^8^ reported that functional connectivity decreases with the worsening of cognitive performance and loss of frontotemporal connectivity may be a promising biomarker of cognitive impairment in PD, and Arroyave et al.^9^ suggested that PD had lower coherence of intra- and inter-hemispheric functional connectivity in the alpha frequency band compared with healthy controls. The common limitation in these studies is that they cannot be automatically used to identify PD patients at the individual level. The development of machine learning provides ideas and approaches to our current limitation. We intend to use the most basic classifier (support vector machine, SVM) to complete the task of identifying early PD patients by learning the characteristic parameters of the brain network. However, how to extract the characteristics of brain network for identifying early PD is the most critical challenge. We will elaborate the basis and advanced improvement of the brain network characteristic indicators adopted in this paper from the following aspects.

During the past decade, the properties of brain functional connectivity network based on EEG has been extensively studied using the sliding window method. A fixed window-size is usually selected heuristically, since no consensus exists yet on the choice of the optimal window-size. Moreover, without a known ground-truth, the validity of the computed properties of brain functional connectivity network remains questionable and unclear. Brain activity is thought to consist of a series of alternating transient steady-state patterns^10–12^. Constructing the EEG data segments corresponding to each transient steady-state pattern of brain activity into a brain functional connectivity network is expected to become a theoretical method for constructing a brain network. In the analysis of brain activity with resting EEG, the transient steady-state pattern at the minimum time scale that can be recognized is described as the EEG microstate^13^. The process of brain activity can be described by a series of alternating and limited microstate sequences of scalp electric field distribution in the resting state^14,15^. Each microstate sequence lasts for a short period of time (tens of milliseconds), and then rapidly changes into another, which remains stable for tens of milliseconds of time^13^. Moreover, studies have shown that the various classes of EEG microstates are determined by corresponding specific brain networks^16,17^. Therefore, the improved data-driven brain network construction based on microstate segmentation proposed in this work can improve the existing network partitioning schemes.

In our previous study, we demonstrated that a unique EEG microstate class and the abnormal EEG microstate features existed in early PD^18^. The abnormality of the microstate properties originates from the complex dynamic brain activity transformation process in which other microstate classes appear between the appearance of one microstate class and its reappearance^14,15^. Moreover, many EEG-based studies have found the changes of dynamic functional connectivity networks in several neurological disorders^10,14,15^, including the motor deficits and cognitive impairment in PD^11,12^. It is reasonable for us to assume that in the brain activity of a specific subject, the brain networks corresponding to each microstate class are dynamically changing, and such dynamic attributes may reflect the pathological essence of the early PD patients. We speculate that the abnormal microstate characteristics of early PD are derived from the abnormal dynamic functional connectivity of brain networks. Therefore, exploring the characteristics of dynamic functional connectivity network constructed based on microstate segmentation is expected to reveal the unique features of brain network in early PD.

Currently, most of the existing studies on dynamic functional connectivity network focus on two aspects: (1) the changing patterns of whole brain network^19^, and (2) temporal properties of functional connectivity between pair of specific brain regions^20,21^. The former may be less sensitive to the identification of local changes in the brain and the latter usually identifies functional connectivity features that are too cumbersome and lack regularity. Actually, studies have shown that the human brain is intrinsically organized into dynamic and spatiotemporal interaction network^22,23^, demonstrating remarkable spatiotemporal variability in its function and structure^23,24^. However, the spatiotemporal properties of dynamic brain networks (e.g., the temporal variability and spatial variability associated with a specific brain region) have never been investigated in resting EEG studies for early PD. In addition, the EEG microstate features also describe the comprehensive characteristics of temporal and spatial properties of brain activity^18^. Hence, temporal and spatial properties of dynamic brain network may convey the important wealth of information and thus help deeper understanding of brain networks in early PD. Intuitively, jointly using temporal and spatial variability of dynamic brain network based on microstate can further improve the performance of early PD diagnosis.

In this paper, we first proposed novel measures based on EEG microstate to characterize the temporal and spatial variability of dynamic functional connectivity network at each specific brain region corresponding to the location of EEG channels. Specifically, we respectively applied (1) sliding window method as baseline (2) microstate sequence method and (3) microstate class method to construct the dynamic functional connectivity network, and calculated their corresponding time-space variability feature sets. Then, we used a common SVM framework to integrate spatiotemporal variability of dynamic functional connectivity network for early PD classification. By comparing the classification accuracy of the three feature sets, it can be determined that the spatiotemporal variability of the dynamic functional connectivity network calculated based on microstate class method has the best effect in identifying early PD. The results indicates that the abnormality of early PD pathological brain network is reflected in the brain network based on microstate class to the greatest extent. Furthermore, we investigated the changing patterns of the temporal variability and spatial variability in early PD patients, and determined the characteristic indicators corresponding to the location of specific brain regions. Finally, we explored the correlation between these characteristic indicators and clinical scale scores to explain the relation between pathology-induced changes of the dynamic functional connectivity network and the motor and cognitive impairments in early PD. The significance of this work is that it can provide a tool for clinical intelligent assisted diagnosis of early PD, and on the other hand, the validity and reliability of the characteristic brain network indicators of early PD can be verified, thereby further revealing the pathological brain network changes of early PD.

## Results

In this section, we present the comparison of classification results among different methods and the further analysis of unique spatio-temporal variability in early PD.

### Classification performance

**Fig. 1:**
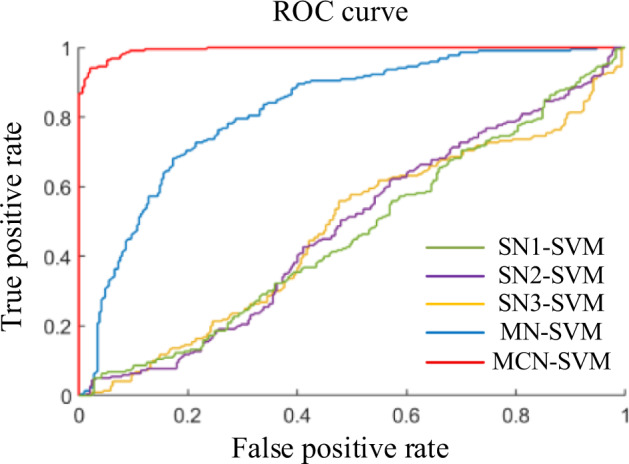
ROC curves of different methods on the early PD vs. HC classification. SN1 = sliding-window-based networks (with window length of 1000, step length of 500); SN2 = sliding-window-based networks (with window length of 1000, step length of 1000); SN3 = sliding-window-based networks (with window length of 500, step length of 500); MN = microstate-window-based networks; MCN = microstate-class-window-based networks.

The classification results on the early PD vs. HC classification task produced by our proposed methods (MN-SVM and MCN-SVM) and other three SN-SVM methods are summarized in Table 1 and Fig. 1. As observed, microstate-based methods perform significantly better classification performance than the traditional sliding window algorithm (regardless of the sliding window parameter configuration, the classification effect is similar), indicating that the brain networks constructed according to the distribution characteristics of microstates can reflect the activity characteristics of the brain in diseased or healthy states in a tiny time period. The validity and value of the brain network construction algorithm based on a priori microstate theory is also reflected. Moreover, the MCN-SVM classifier consistently achieves the best performance compared with other methods. Specifically, MCN-SVM achieves the accuracy of 95.69 ± 0.0636, the sensitivity of 92.97 ± 0.1136, the specificity of 98.41 ± 0.0515, F1 score of 93.67 ± 0.0954 and AUC of 0.99 ± 0.0002, indicating the effectiveness of our proposed algorithm in early PD diagnosis.

**Table 1:** Comparison of different methods for early PD vs. HC classification.

Method	Accuracy (%)	Sensitivity (%)	Specificity (%)	F1 Score	AUC
SN1-SVM	51.05 ± 0.1551	42.30 ± 0.2667	60.21 ± 0.1874	38.63 ± 0.2164	0.50 ± 0.0018
SN2-SVM	49.49 ± 0.1684	37.95 ± 0.2553	61.11 ± 0.1924	36.40 ± 0.2139	0.49 ± 0.0018
SN3-SVM	49.96 ± 0.1435	40.11 ± 0.2222	60.73 ± 0.2054	37.50 ± 0.1810	0.48 ± 0.0018
MN-SVM	69.27 ± 0.1503	41.83 ± 0.2254	92.81 ± 0.1294	50.74 ± 0.2182	0.86 ± 0.0015
MCN-SVM	95.69 ± 0.0636	92.97 ± 0.1136	98.41 ± 0.0515	93.67 ± 0.0954	0.99 ± 0.0002

Significant differences are displayed in bold and italics type. SD, standard deviation. Adjustment for multiple comparisons: FDR. Significant statistical difference with *p < 0.05 and **p < 0.01.

### Spatiotemporal variability of brain microstate-class-window-based networks

The above studies indicate that the brain network constructed based on microstates can be divided into four brain network sets according to the microstate classes and the temporal variability and the spatial variability of the brain networks corresponding to each microstate class are calculated as the classification features, which can most accurately identify early PD patients. Therefore, exploring the characteristics of spatiotemporal variability of brain microstate-class-window-based networks in early PD can provide value insights for the pathological brain network abnormalities of early PD.

### Temporal variability of brain regions in microstate networks

**Fig. 2:**
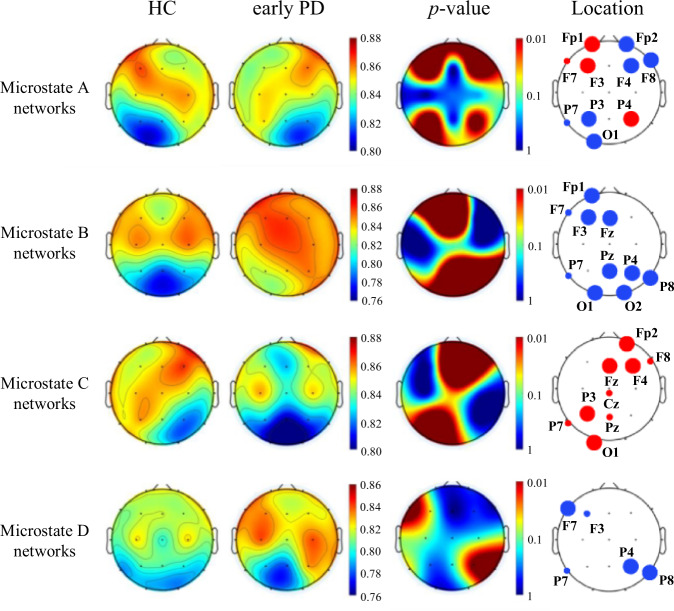
Topological distribution of temporal variability. The red points indicate the positions in which the temporal variability of microstate functional connectivity networks in healthy controls are obviously higher than that in early PD patients, and the blue points represent the positions that have lower temporal variability in healthy controls. The large points represent significant group difference (*p* < 0.01, FDR corrected), and the small points indicate *p* < 0.05 with FDR corrected.

First, we explored the temporal variability of brain regions in four microstate brain networks. Topological distribution of temporal variability of microstate functional connectivity networks (averaged over the subjects) for both groups and their differences in the four-microstate networks was shown in Fig. 2. There were significant differences in the temporal variability of HC and early PD patients in the brain network corresponding to each microstate class.

For the microstate A network, in the HC group, the brain regions with high temporal variability were mainly located in the left frontal lobe, while the brain regions with low temporal variability were mainly located in the left occipital lobe. In contrast, the temporal variability distribution of microstate A network in early PD patients was opposite, with high temporal variability in the right frontal lobe and low temporal variability in the right occipital lobe with. The regions with main differences were the left frontal and right parietal lobe (HC > early PD); right frontal, left parietal, and left occipital lobe (HC < early PD). In the microstate B network, the temporal variability of most brain regions in early PD patients was significantly higher than that in healthy subjects. The regions with main differences were the left frontal, parietal, and occipital lobe. Compared with the microstate B network, the phenomenon in the microstate C network was just the opposite. The temporal variability of most brain regions in early PD patients was significantly lower than that in healthy controls, and the regions with main differences were the right prefrontal, frontal, and left occipital lobe. Similarly, the temporal variability of microstate D network was significantly higher in early PD group than in HC group, mainly distributed in left frontal and right parietal lobe.

### Spatial variability of brain regions in microstate networks

**Fig. 3:**
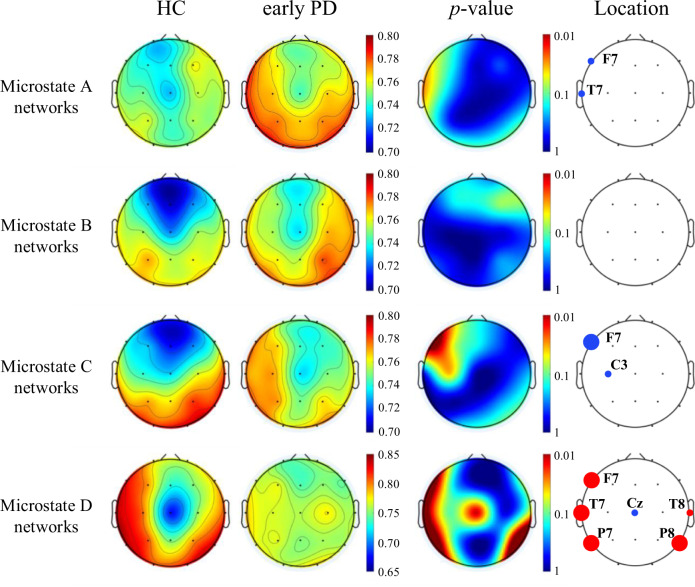
Topological distribution of spatial variability of microstate functional connectivity networks (averaged over the subjects) for both groups and their differences in the four-microstate networks. The red points indicate the positions in which the spatial variability of microstate functional connectivity networks in healthy controls are obviously higher than that in early PD patients, and the blue points represent the positions that have lower spatial variability in healthy controls. The large points represent significant group difference (*p* < 0.01, FDR corrected), and the small points indicate *p* < 0.05 with FDR corrected.

Compared with the significant differences in the temporal variability of brain regions in the early PD and HC groups, the differences in the spatial variability of brain regions in the four microstate networks were much less in the early PD and HC groups. As shown in Fig. 3, the spatial variability of brain regions in microstate A network and microstate B network in early PD patients was generally higher than that in healthy subjects, but the differences between groups were similar, and only exist in a small area of brain regions in the left frontal and left temporal lobe of PD microstate A network. For microstate C network, the spatial variability of HC group was more regular and different, showing that the spatial variability of the frontal lobe was significantly less than that of the occipital lobe, while the spatial variability of early PD group was more uniform, and the significant difference between HC group and early PD group was distributed in the left frontal lobe. A similar phenomenon was found in the microstate D network of healthy subjects, that is, the spatial variability of the brain regions was more regular and significantly different. The spatial variability of the central region was significantly less than that of the left frontal lobe, temporal lobe, and the left and right parietal lobes, while the spatial variability of early PD group was more uniform. Besides, the left frontal, bilateral temporal and left-right parietal lobe showed significant differences in spatial variability between the two groups.

### Temporal and spatial variability of the whole brain in microstate networks

**Fig. 4:**
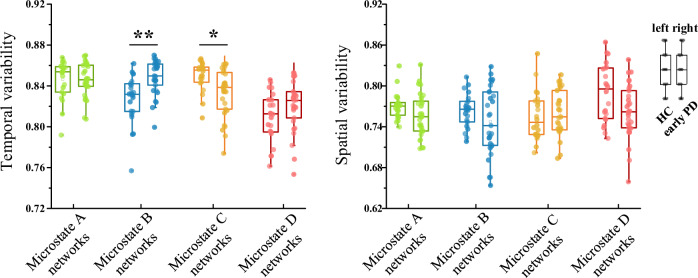
The temporal and spatial variability of whole brain microstate functional connectivity networks in patients with early PD and healthy controls, respectively. The green, blue, yellow, and red boxes represent functional connectivity networks of microstate class A–D, respectively. The box plot on the left of each pair represents healthy controls and the box plot on the right represents patients with early PD. * indicates a statistical difference with *p* < 0.05 and ** indicates a statistical difference with *p* < 0.01. FDR correction has been performed for *p*-values. Each dot re*p*resents every single subject. The horizontal line within the box indicates the median, box limits represent the upper and lower quartiles, and the whiskers indicate the range of values within 1.5 times the interquartile range.

The characteristic value of the whole brain variability can be obtained by averaging the characteristic value of each brain region of each subject. Figure 4 shows the temporal and spatial variability of whole brain microstate functional connectivity networks (averaged among the subjects) for both groups. The temporal variability of microstate B network and microstate C network was significantly different between early PD and HC groups. As shown in Table 2, the time variability of microstate B network in early PD patients was significantly higher than that in healthy subjects (*t* = −3.762, *p* = 0.002), while the time variability of microstate C network was significantly lower (*t* = 2.809, *p* = 0.014). This also corresponded to the differential distribution of temporal variability in brain regions as shown in Fig. 3. As for spatial variability, the right side of Fig. 4 indicated that there were no significant inter-group differences between early PD patients and healthy subjects (Table 3).

**Table 2:** Descriptive statistics of temporal variability of whole brain microstate functional connectivity networks in patients with early PD and healthy controls.

	HC subjects		Early PD patients		Group comparisons	
	Mean	(SD)	Mean	(SD)	*t*-value	*p*-value
Microstate A networks	0.85	0.02	0.85	0.02	0.042	0.966
Microstate B networks	0.83	0.02	0.85	0.02	−3.762	0.002**
Microstate C networks	0.85	0.01	0.83	0.02	2.809	0.014*
Microstate D networks	0.81	0.02	0.82	0.03	−1.648	0.141

Significant differences are displayed in bold and italics type. *SD* standard deviation. Adjustment for multiple comparisons: FDR. Significant statistical difference with *p < 0.05 and **p < 0.01.

**Table 3:** Descriptive statistics of spatial variability of whole brain microstate functional connectivity networks in patients with early PD and healthy controls.

	HC subjects		Early PD patients		Group comparisons	
	Mean	(SD)	Mean	(SD)	*t*-value	*p*-value
Microstate A networks	0.77	0.02	0.76	0.03	1.665	0.205
Microstate B networks	0.76	0.02	0.75	0.05	1.368	0.237
Microstate C networks	0.76	0.04	0.76	0.03	−0.541	0.591
Microstate D networks	0.79	0.04	0.76	0.04	2.330	0.096

Significant differences are displayed in bold and italics type. SD, standard deviation. Adjustment for multiple comparisons: FDR. Significant statistical difference with *p < 0.05 and **p < 0.01.

### Clinical correlations with the temporal and spatial variabilities of brain regions

**Fig. 5:**
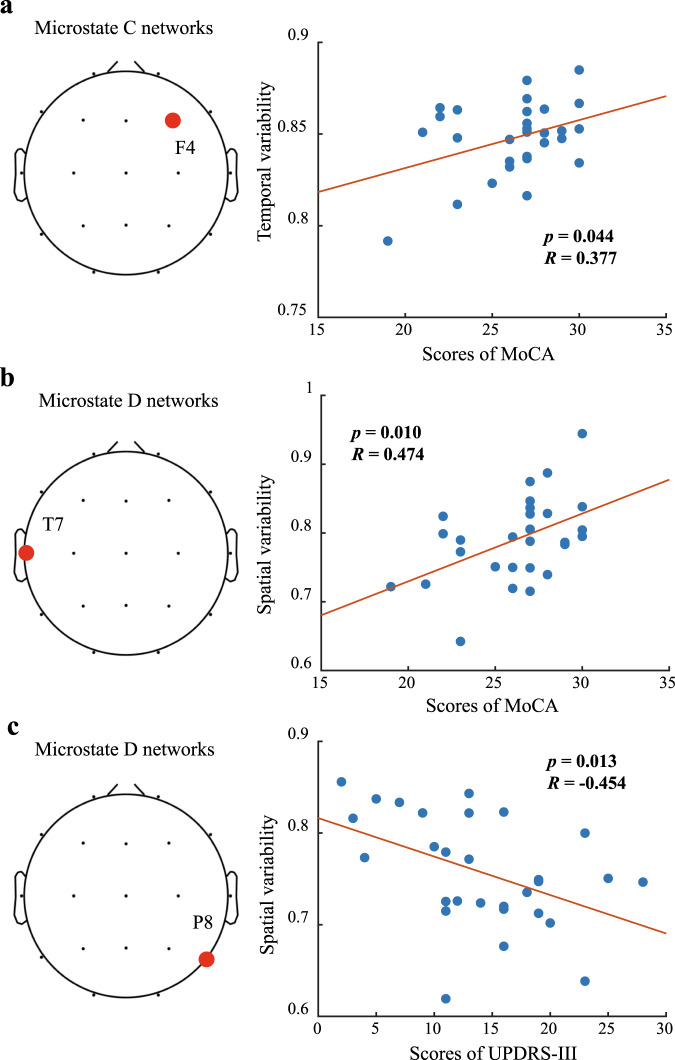
Spearman’s correlation between scores of clinical scales and spatiotemporal variabilities of local scalp regions in microstate networks. **a** Spearman’s correlation between scores of MoCA and temporal variability of local scalp region at electrode channel ‘F4’. **b** Spearman’s correlation between scores of MoCA and spatial variability of local scalp region at electrode channel ‘T7’. **c** Spearman’s correlation between scores of UPDRS-III and spatial variability of local scalp region at electrode channel ‘P8’. The red point indicates the region in which the temporal and spatial variability of microstate brain networks in healthy controls are obviously higher than that in early PD patients. FDR correction was performed for *p*-values.

As shown in Fig. 5a, temporal variability of both the right frontal lobe of the microstate C network was significantly negatively correlated with the MoCA scale (*R* = 0.377, *p* = 0.044). In addition, the temporal variability of the frontal lobe in microstate C network of healthy subjects was significantly higher than that in patients with early PD (Fig. 2). It suggested that temporal variability of the frontal lobe in microstate C network increases with the improvement of cognitive level.

Spatial variability of microstate D network in early PD patients was significantly correlated with clinical scale scores, and as shown in Fig. 5b, spatial correlation of left temporal lobe in early PD patients was significantly positively correlated with MoCA scale (*R* = 0.474, *p* = 0.010). Besides, the spatial variability of microstate D network in the left temporal lobe of healthy subjects was significantly higher than that of patients with early PD (shown in Fig. 3). It indicated that the higher the spatial variability of the microstate D network in the left temporal lobe, the better the cognitive level of early PD patients. In addition, the spatial correlation of the right occipital lobe in early PD patients was significantly negatively correlated with the UPDRS-III scale (*R* = −0.454, *p* = 0.013) as shown in Fig. 5c. The spatial variability of the microstate D network in the right occipital lobe of patients with early PD was significantly lower than that of healthy subjects (shown in Fig. 3), indicating that the decreased spatial variability of microstate D network in the right occipital lobe was associated with dyskinesia symptoms in early PD patients.

## Discussion

Recently, numerous studies^25–29^ suggest that the psychiatric and neurological disease exhibit significant changes in dynamic brain network properties. Quantification and application of changed dynamics in brain functional connectivity networks may contribute to a better understanding of brain diseases, and ultimately provide better prognostic indicators or auxiliary diagnostic tools. At present, most studies are focused on the properties of brain functional connectivity network constructed by sliding window method. However, few studies have explored evidence-based brain network construction algorithms that reflect disease specificity. In this work, we first define a novel approach to characterize the spatiotemporal variability of dynamic functionally connectivity networks based on EEG microstates, and further develop a classification framework for integrating spatiotemporal variability of brain networks to improve early PD diagnostic performance. The experimental results suggest that our proposed method outperform the conventional method based on sliding window, indicating the advancement of brain network construction method based on EEG microstates for identifying the abnormalities of pathological brain network in early PD. Moreover, our work also confirms that temporal and spatial properties of brain interaction patterns is effective in boosting the diagnosis performance of early PD. It is worth noting that spatio-temporal variability can be potentially used for analyzing the fundamental properties of brain network or brain activity, e.g., the underlying relationship between spatiotemporal variability of a specific brain region and its neural activity and structural connectivity. The temporal and spatial variability calculated by our proposed method are relevant to the pathological changes in the brain of early PD.

In this work, our research objective is to explore whether the spatial and temporal variability characteristics of dynamic brain networks based on EEG microstates can effectively identify early PD patients compared with the conventional sliding window method. To address this, a classifier with fixed parameters and relatively simple learning ability is needed for each type of input feature data, so that the accuracy of classification comes from the contribution of the differences between the input characteristic parameters. In addition, the data samples included in this work are small, so the binary task of processing small sample data has become the selection criterion of the classifier in this work. Although there are various selection and optimization strategies for machine learning classifiers at present, according to the objective of this study, standard SVM with default parameters configuration can meet the above requirements. Therefore, we adopted standard SVM with linear kernel, and determined the type of feature parameters with the best classification effect by replacing different types of input feature sets. Due to the limited data amount of clinical data collected, our next research plan is to collect more data to verify the generalization potential of the algorithm and the features proposed in this paper. In addition, we will further collect clinical scale scores of patients (including assessment of motor and cognitive function) to verify the clinical correlation of such characteristic biomarkers.

**Fig. 6:**
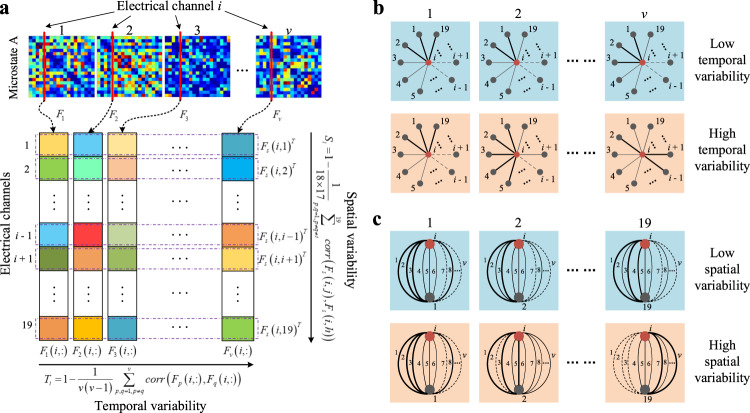
Illustration of spatiotemporal variability algorithm with microstate A networks as an example. **a** Illustration of computational processes for temporal variability and spatial variability of specific scalp location (electrical channel *i*). **b** The schematic of temporal variability of the functional architecture. **c** The schematic of spatial variability of the functional architecture. The solid line width represents the strength of the functional connectivity between two regions (electrical channel’s scalp location) and the dashed line represents fairly weak functional connectivity. *v* indicates the number of microstate A networks.

The temporal variability of each scalp region characterizes the connectivity pattern changes of all functional connectivity between this region and other scalp regions over time. As shown in Fig. 6b high temporal variability reveals that the dynamical properties of a connectivity sequence of functional connectivity between one scalp region and other scalp regions are independent, that is, the functional modules (*i.e*., functional connectivity patterns) consisting of functional connectivity between a given scalp region and other scalp regions are relatively independent in each time window. In contrast, low temporal variability indicates that the functional architecture of a given scalp region is highly correlated within each time window, *i.e*., the connectivity series of dynamical functional connectivity between this scalp region and all other scalp regions are highly synchronized. The large time variability indicates the high flexibility of network nodes and the strong instability of the whole network. In the set of brain networks corresponding to a specific microstate class, the time variability of the whole brain is high, indicating that each time the transient steady state brain activity reflected by scalp electrical activity occurred, the corresponding brain network had relatively independent functional connectivity modules, and the functional connectivity pattern of the brain network is more unstable.

In this work, we have found that microstate B networks are more flexible in early PD than that of healthy controls. Moreover, our results suggest that the activity of this functional connectivity pattern in early PD is mainly reflected in the occipital lobe. Previous studies have shown that microstate class B expresses the visual network^16,30,31^, and the occipital lobe also maps the visual network^32^. Therefore, we speculate that the fluctuation of the visual network of early PD patients in a short period of time is stronger than that of healthy controls, which may be the manifestation of visual impairment (such as rapid eye movement) in early PD patients. Our results suggest that the global brain network corresponding to microstate class C in early PD is more stable than that of healthy controls, and from the perspective of local brain regions, this significantly stable functional connectivity pattern is mainly distributed in the frontal lobe, and it is significantly positively correlated with the cognitive level of early PD patients. It indicates that when early PD patients’ brain activity appears microstate class C, the more inflexible the frontal lobe is in the process of dynamic changes of functional connectivity pattern, the worse the cognitive level of patients is. Previous studies have revealed that the saliency network corresponding to microstate class C can reflect the cognitive level of PD patients^18^, and the frontal lobe maps the cognitive function^33^, which supports our findings.

The spatial variability of each scalp region is the change in the synchronicity of interruption and connectivity patterns of all functional connectivity between this region and other scalp regions. As shown in Fig. 6c high spatial variability means that the interruption and connectivity patterns of the time series of functional connectivity between a given scalp region and other scalp regions are independent. On the contrary, low spatial variability means that the pattern of functional connectivity and interruption between a given scalp region and other scalp regions is highly correlated during network changes, *i.e*., the time series of dynamical functional connectivity between this scalp region and all other scalp regions are highly synchronized.

The locations of the characteristic brain regions that determine the spatial variability of the functional connectivity network corresponding to the microstate D in early PD are significantly lower than that of the healthy controls were the left frontal lobe, bilateral temporal lobe, left posterior parietal lobe, and right posterior parietal lobe. This indicates that the dynamic synchronization of functional connectivity between the above brain regions and other regions of early PD is significantly higher than that of healthy controls. The results of this study further reflect that the spatial variability of the left temporal lobe in the microstate D network of early PD patients is significantly lower than that of healthy subjects and positively correlated with the cognitive level of patients. This suggests that when the patients have specific microstate D in brain activities, the stronger the dynamic synchronization of functional connectivity between the left temporal lobe and other regions (*i.e*., the smaller the spatial variability), the worse the cognitive level of the patients. Besides, the function of temporal lobe has a certain relationship with memory and emotion^34^. Previous studies have shown that the temporal lobe is the response region of cognitive impairment in Alzheimer’s disease and even dementia, and it reflects the cognitive function network^35,36^. Therefore, this work can further infer that the spatial variability of microstate D network in the left temporal lobe can effectively reflect the cognitive level of PD patients. Furthermore, in the microstate D networks of early PD patients, the spatial variability of right posterior parietal lobe is significantly lower than that of healthy subjects and significantly negatively correlated with the level of motor function of the patients. It means that when PD patients have specific microstate D in brain activities, the stronger the dynamic synchronization of functional connectivity between the right posterior parietal lobe and other regions (*i.e*., the smaller the spatial variability), the worse the patients’ motor symptoms will be. Previous studies have confirmed that the parietal lobe can reflect the motor function network^37,38^, which further supports our findings.

There are several potential limitations to this study. First, more subjects need to be recruited to provide more evidence for studying the correlation between temporal and spatial dynamic characteristics of microstate network and motor function/cognitive function, and to further verify the reliability of characteristic indicators of specific changes. Second, the dynamic temporal and spatial variability of microstate functional networks should be further explored and validated using the fMRI-EEG simultaneous recording technology. Finally, magnetoencephalography (MEG) (with more spatial information) experiment should be executed to provide more evidence to explain the dynamic variability of the early PD microstate brain networks.

## Methods

### General information about participants

In this study, a total of 29 drug-off early PD patients (9 males, 20 females, age: 62.4 ± 6.3 years old) were recruited from the department of Neurology, General Hospital of Tianjin Medical University, and 22 age-matched healthy control (HC) subjects (11 males, 11 females, age: 63.8 ± 5.5 years old) as the HC group. All patients with early PD were screened by the same neurologist using the same criteria (inclusion criteria: patients with the Hoehn and Yahr rating scale (H-Y) stage: 1). In addition, none of the healthy subjects had a history of neurological or psychiatric illness. The inclusion criteria for patients with early PD were as follows: 1. all participants were diagnosed with primary PD; 2. no head tremor symptom; 3. all patients with early PD were stopped from medication for more than 12 h before EEG collection; 4. no history of psychiatric disorders; 5. no history of head trauma with loss of consciousness. The detailed information of included subjects is shown in Table 4.

**Table 4:** Subject characteristics.

	HC subjects (*N* = 22) (*N* = 22)	Drug-off early PD patients (*N* = 29)
Age (years, mean ± SD)	63.8 ± 5.5	62.4 ± 6.3
Sex (Male/Female)	11/11	9/20
H & Y stage	n.a.	1
Scores of UPDRS-III	n.a.	15.8 ± 7.5
Scores of MoCA	n.a.	26.2 ± 2.9

H&Y stage = Hoehn and Yahr rating scale, UPDRS-III = the third part of the Unified Parkinson’s Disease Rating Scale, MoCA = Montreal Cognitive Assessment, n.a. = not applicable.

### Acquisition protocol

All participants (including early PD patients and healthy controls) seated comfortably in a quiet semi-dark room with eyes closed but kept awake. EEG signals were recorded between 9 a.m. and 11 a.m.. EEG activities of the brain scalp at 19 electrodes were collected. Besides, additional channels were also used to monitor four electrooculograms (EOGs), as well as electrocardiogram (ECG) and electromyogram (EMG) for further preprocessing. All electrodes used for collection are kept below 5 kΩ in impedance. EEG signals were sampled at 500 Hz. EEG data of each participant was monitored lasting for 15 to 20 min.

All patients received a detailed behavioral and neuropsychiatric assessment. The evaluation scales used in the present study are Uniform Parkinson’s Disease Rating Scale-III (UPDRS-III) and Montreal Cognitive Assessment (MoCA). UPDRS-III and MoCA are used as a quantitative criterion to assess patients with dyskinesia^39^ and cognitive impairment^40^, respectively. All subjects understood the purpose of collecting the data and the significance of the study, and signed the informed consent.

The studies involving human participants were reviewed and approved by Medical Ethics Committee of Tianjin Medical University General Hospital. All subjects understood the purpose of collecting the data and the significance of the study, and signed the written informed consent form.

### Analysis materials

Nineteen Ag/Agcl scalp electrodes (active electrodes, SYMTOP, Beijing, China) Fp1, Fp2, F3, F4, C3, C4, P3, P4, O1, O2, F7, F8, T7, T8, P7, P8, Fz, Cz and Pz were placed on the scalp in accordance with international standard 10–20. These 19 Ag/AgCl scalp electrodes are linked to 19 data channels, and 2 reference electrodes (A1 and A2) are linked to the both earlobes. The UEA-BZ amplifier (SYMTOP, Beijing, China) was linked to these data channels to amplify and digitize the EEG signals. The acquisition of EEG signals was handed by Study Rome software. The preprocessing of raw EEG was performed in MATLAB software (MathWorks Inc., Natick MA, United States). EEG microstate analyses were carried out by Cartool software (the Key Institute for Brain-Mind Research, Zurich, Switzerland). Statistical analyses were performed by SPSS 25.0 software (IBM Inc., Chicago, IL, United States) and MATLAB software (MathWorks Inc., Natick MA, United States).

### EEG preprocessing

Fast Independent Component Analysis (Fast-ICA) was applied on the continuous EEG signal^41^. The correlation components of horizontal and vertical eye artifacts, ECG, and EMG artifacts were set to zero to reconstruct the EEG signals after the removal of artifacts. Then, based on existing research^18^, each EEG signal was processed by a 2–20 Hz band-pass Finite Impulse Response (FIR) filter to eliminate the interference of high frequency noises. However, artifacts caused by body movement and technical artifacts were difficult to be removed by Fast-ICA and filtering, such that the epochs of EEG data that were contaminated due to the presence of artifacts were rejected by manual screening. Successive EEG recordings for a total of 5 min of each subject were retained for microstate analyses.

### Functional connectivity network construction based on EEG microstate analysis

In order to obtain the microstate sequences of each subject, the preprocessed EEG signals were imported into Cartool software for EEG microstate analysis. Global field power (GFP) is a measure of scalp potential intensity, and is based on potential differences between the potential of all electrodes at each sampling point and their average potential, leading to a scalar value of the field intensity at each sampling point:^42^

1 $$GFP\left( t \right) = \sqrt {\frac{{\mathop {\sum}\limits_{i = 1}^n {\left[ {u_i\left( t \right) - \bar u\left( t \right)} \right]^2} }}{n}}$$

here, *n* represents the number of electrodes, *u*_*i*_ (*t*) denotes the measured potential strength of the *i* th electrode at time *t*, and $\bar u\left( t \right)$ represents the average potential strength of all electrodes at time *t*. Since the highest signal-to-noise ratio of EEG is located at the peak of GFP, the EEG topographic maps at the time point of GFP peaks were selected for clustering analysis. The clustering algorithm called modified K-means algorithm^43^ was performed to obtain respective four microstate classes of early PD patients and healthy controls, respectively. Then the generated microstate class-labeled maps were used as templates to assign original continuous EEG signals of each participant to homologous microstate classes (A, B, C or D class), thus a microstate sequence corresponding to EEG sequence was formed.

**Fig. 7:**
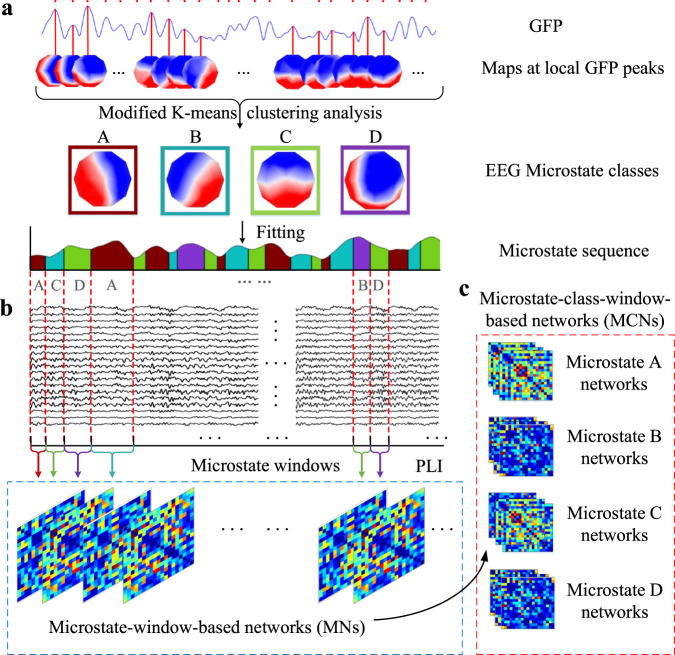
Functional connectivity network construction based on EEG microstate analysis. **a** EEG microstate analysis is used to form the microstate sequence corresponding to EEG sequence. **b** The microstate windows are used to construct functional connectivity networks based on microstate sequences. **c** The functional connectivity networks based on microstate classes. GFP global field power, SVM support vector machine, PLI phase lag index.

We used the segmented microstate windows in the microstate sequence to construct the functional connectivity network, as shown in Fig. 7. Specifically, for each subject, we first divided the EEG sequence into *n* non-overlapping microstate windows according to the distribution of each microstate class in the microstate sequence, and the EEG data corresponding to each window has a specific microstate class. Then, we adopted the phase lag index (PLI) algorithm^44^ to obtain the brain functional connectivity network (*i.e*., adjacency matrix) between 19 detection electrodes of each subject. The functional connectivity of pair-wise EEG channels within the *r*th microstate window can be obtained by quantify the asymmetry of the phase difference distribution between two signals, and it can be defined as:

2 $$F_r\left( {i,j} \right) = \left| {\left\langle {sign\left[ {\sin \left( {\Delta \phi \left( {t_k} \right)} \right)} \right]} \right\rangle } \right|,k = 1,2,...,m_r,i \,\ne\, j$$

where *F*_*r*_(*i, j*) defines the functional connectivity of electrical channel *i* and *j* within the *r*th microstate window,$\left\langle . \right\rangle$denotes the mean value inside, Δ*ϕ*(*t*_*k*_) is the phase difference calculated at *k* time points and *m*_*r*_ is the number of time points within the *r*th microstate window. The range of PLI values is from 0 to 1, with 0 standing for no coupling or coupling with zero lag between the two signals and 1 indicating perfect (non-zero delay) phase locking. Finally, the 19 × 19 adjacency matrix containing PLI values of all pair-wise EEG channels can be taken as the functional connectivity network *F*_*r*_ within the *r*th microstate window. Thus, as shown in Fig. 7b, a set of functional connectivity networks are obtained with *v* microstate windows (called microstate-window-based networks, MNs), *i.e*., $F = \left\{ {F_1, \cdot \cdot \cdot ,F_r, \cdot \cdot \cdot ,F_v} \right\}$. Furthermore, we divide the functional connectivity networks corresponding to each microstate classes into separate sets of brain networks (called microstate-class-window-based networks, MCNs) as shown in Fig. 7C.

### Temporal and spatial variabilities of dynamic functional connectivity network

Figure 6a illustrates the computational processes of temporal and spatial variability of specific scalp location (electrical channel *i*). Given the set of functional connectivity networks, $F = \left\{ {F_1, \cdot \cdot \cdot ,F_r, \cdot \cdot \cdot ,F_v} \right\}$, to characterize the temporal variability of dynamic functional connectivity network at a specific scalp location (electrical channel location), the temporal variability of dynamic functional connectivity associated with a specific scalp location (electrical channel *i*) is defined as:

3 $$T_i = 1 - \frac{1}{{v\left( {v - 1} \right)}}\mathop {\sum}\limits_{p,q = 1,p \ne q}^v {corr\left( {F_p\left( {i,:} \right),F_q\left( {i,:} \right)} \right)}$$

where *F*_*p*_(*I*,:) represents the functional architecture of electrical channel *i* at the *p*th microstate window (e.g., when *i* = 5, $F_p\left( {5,:} \right) = \left[ {F_p\left( {5,1} \right),F_p\left( {5,2} \right), \cdot \cdot \cdot ,F_p\left( {5,4} \right),F_p\left( {5,6} \right), \cdot \cdot \cdot ,F_p\left( {5,19} \right)} \right]^T$) and $corr\left( {F_p\left( {i,:} \right),F_q\left( {i,:} \right)} \right)$ is the correlation coefficient between two different functional architectures measuring their temporal similarity.

In order to characterize the spatial variability of dynamic functional connectivity network at the specific scalp location, firstly, the changing profile of functional connectivity between a pair of electrical channels within all microstate windows is defined as a spatial functional connectivity sequence (e.g., $F_s\left( {i,j} \right) = \left[ {F_1\left( {i,j} \right),F_2\left( {i,j} \right), \cdots ,F_v\left( {i,j} \right)} \right]^T$ denotes the spatial functional connectivity sequence between electrical channel *i* and *j*). Then the spatial variability of dynamic functional connectivity associated with a specific scalp location (electrical channel *i*) is defined as:

4 $$S_i = 1 - \frac{1}{{18 \times 17}}\mathop {\sum}\limits_{j,h = 1,j \ne h \ne i}^{19} {corr\left( {F_s\left( {i,j} \right),F_s\left( {i,h} \right)} \right)}$$

where $corr\left( {F_s\left( {i,j} \right),F_s\left( {i,h} \right)} \right)$ denotes the correlation coefficient between two different spatial functional connectivity sequences *F*_*s*_(*i, j*) and *F*_*s*_(*i, h*), such that it measures the spatial similarity of all functional connectivity sequences associated with a specific scalp location (electrical channel *i*).

In general, two types of variabilities defined at a particular scalp location reflect the local variability of dynamic functional connectivity networks from two different perspectives. Specifically, the temporal variability defined in Eq. (3) reflects the time-varying characteristics of the functional structure of local brain regions, and the spatial variability defined in Eq. (4) embodies the space-varying characteristics of spatial functional connectivity sequences associated with local brain regions.

### Methods for comparison

**Fig. 8:**
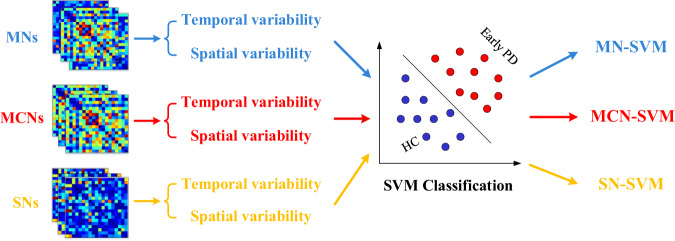
Features calculated by different methods are taken in a common SVM classifier for disease classification.

In this work, we designed three kinds of features for the disease classification based on standard SVM classifier with linear kernel (with default parameters). The differences in classification effectiveness depend on the attributes of the features that are inputs of the SVM. The spatiotemporal variability (temporal variability and spatial variability) of functional connectivity networks based on equal length sliding windows (called sliding-window-based networks, SNs) are considered as the baseline features. The other two sets of features for comparison are the spatiotemporal variability of MNs and MCNs, respectively. We define the three feature classifiers as SN-SVM, MN-SVM and MCN-SVM (Fig. 8). In order to avoid the contingency of the sliding windows, we compared the classification result of SN-SVMs with several popular window lengths and step lengths (with window length of 500, step length of 500; with window length of 1000, step length of 500; with window length of 1000, step length of 1000). In the experimental process of using each feature classifier, two classification tasks (early PD vs. HC) are conducted using a 5-fold cross-validation strategy. Specifically, the whole subject samples (the spatiotemporal variability of 19 EEG channels of 51 subjects (29 early PD vs. 22 HC) calculated from each kind of networks) are randomly partitioned into 5 subsets, from which 4 subsets were randomly selected for training and the remaining subset for testing during each cross-validation process. The above procedure was repeated 10 times and the average of these 50 trails was reported to avoid any bias caused by the partition. We evaluate the classification performance of each method by computing the performance measurements including accuracy, sensitivity, specificity, F1 Score, and the area under receiver operating characteristic (ROC) curve (AUC).

Based on the performance of feature classifiers, we can determine that the features based on which the classification effectiveness is best express distinct abnormal indicator of dynamic brain networks in early PD. Therefore, we further analysis the unique spatiotemporal variability in early PD based on the brain network construction rule corresponding to the best disease classification performance.

### Statistical analysis

The independent-sample *t*-test was performed to evaluate the differences between early PD patients and healthy controls at a *p* < 0.05 significance level. Spearman’s correlation analysis was performed to verify the certain correlation between the indicators and clinical scales, and the correlation was accepted at *p* < 0.05. We used the false discovery rate (FDR) to correct the results.

### Reporting summary

Further information on research design is available in the Nature Research Reporting Summary linked to this article.

## Supplementary information

Reporting Summary

## Data Availability

The anonymized datasets used in this study are available on request from the corresponding authors.

## Code availability

The sample data and all the code that implements the results of this work can be found on Github (https://github.com/ChunguangChu/Spatiotemporal-variability-analysis-based-on-EEG-microstate-network.git).
